# Transcription and Expression of *Plasmodium falciparum* Histidine-Rich Proteins in Different Stages and Strains: Implications for Rapid Diagnostic Tests

**DOI:** 10.1371/journal.pone.0022593

**Published:** 2011-07-22

**Authors:** Joanne Baker, Michelle L. Gatton, Jennifer Peters, Mei-Fong Ho, James S. McCarthy, Qin Cheng

**Affiliations:** 1 Department of Drug Resistance and Diagnostics, Australian Army Malaria Institute, Gallipoli Barracks, Enoggera, Queensland, Australia; 2 Malaria Drug Resistance and Chemotherapy Laboratory, Queensland Institute of Medical Research, Brisbane, Queensland, Australia; 3 Clinical Tropical Medicine Laboratory, Queensland Institute of Medical Research, Brisbane, Queensland, Australia; 4 School of Medicine, University of Queensland, Brisbane, Australia; 5 School of Population Health, University of Queensland, Brisbane, Australia; Burnet Institute, Australia

## Abstract

**Background:**

Although rapid diagnostic tests (RDTs) for *Plasmodium falciparum* infection that target histidine rich protein 2 (PfHRP2) are generally sensitive, their performance has been reported to be variable. One possible explanation for variable test performance is differences in expression level of PfHRP in different parasite isolates.

**Methods:**

Total RNA and protein were extracted from synchronised cultures of 7 P. falciparum lines over 5 time points of the life cycle, and from synchronised ring stages of 10 falciparum lines. Using quantitative real-time polymerase chain reaction, Western blot analysis and ELISA we investigated variations in the transcription and protein levels of pfhrp2, pfhrp3 and PfHRP respectively in the different parasite lines, over the parasite intraerythrocytic life cycle.

**Results:**

Transcription of pfhrp2 and pfhrp3 in different parasite lines over the parasite life cycle was observed to vary relative to the control parasite K1. In some parasite lines very low transcription of these genes was observed. The peak transcription was observed in ring-stage parasites. Pfhrp2 transcription was observed to be consistently higher than pfhrp3 transcription within parasite lines. The intraerythrocytic lifecycle stage at which the peak level of protein was present varied across strains. Total protein levels were more constant relative to total mRNA transcription, however a maximum 24 fold difference in expression at ring-stage parasites relative to the K1 strain was observed.

**Conclusions:**

The levels of transcription of *pfhrp2* and *pfhrp3*, and protein expression of PfHRP varied between different *P. falciparum* strains. This variation may impact on the detection sensitivity of PfHRP2-detecting RDTs.

## Introduction

The ability to accurately diagnose malaria infections is critical to the control and elimination of this disease [1]. This is of particular importance in settings where laboratory facilities are poorly resourced. Rapid diagnostic tests (RDTs) for malaria have the potential to significantly improve malaria diagnosis and to contribute to reduction in morbidity and mortality in endemic countries, particularly in remote areas. When deployed in conjunction with artemisinin-combination therapy and insecticide-treated bednets, they form a cornerstone of current efforts to eliminate malaria [2].

Many of the RDTs marketed to diagnose malaria target *Plasmodium falciparum* histidine-rich protein 2 (PfHRP2) that circulates in the bloodstream of the patient [3]. PfHRP3, another protein generated by the parasite, may also be detected by PfHRP2-detecting RDTs [4]. While PfHRP2-detecting RDTs generally show good sensitivity and specificity, there are also many reported disparities in their performance, with no apparent pattern of geographic or quality-related causative factors [5]. Although most reported sensitivity variations were observed as false negative tests at relatively low parasitemia (< 250 parasites per microlitre, P/µL), in some studies RDTs have been reported to yield false negative results at relatively high parasitemia (> 1000 P/µL) [6].

Several factors may contribute to the variable sensitivity reported for PfHRP2-detecting RDTs. These include genetic variation of PfHRP2 between parasites. We have previously shown that while genetic polymorphism in PfHRP2 is extensive, it does not appear to affect RDT detection sensitivity at levels > 200 P/uL [7]. Another potential factor may be variation in the levels of PfHRP2 and PfHRP3 proteins produced by different parasite strains, likely a consequence of variations in mRNA transcription levels.

PfHRP2 is a 60-105 kDa water-soluble protein specific to *P. falciparum*. It is encoded by *pfhrp2*, a subtelomeric gene located on chromosome 7 [8], [9], [10], [11]. Studies of gene expression across the parasite lifecycle indicate that PfHRP2 protein synthesis begins with immature parasites (rings), two hours after invasion of the red blood cell [9]. Transcription and translation continues throughout the rest of the blood stage lifecycle in asexual parasites and in the developing gametocytes [8], [11], [12], [13]. The protein is found on the surface of infected erythrocytes, within the parasite cytosol and in the peripheral blood of infected individuals [14]. The protein has multiple copies of very similar alanine and histidine-rich repeats [8] that serve as suitable epitopes for recognition by antibodies present in purpose-designed antigen-capture immunochromatographic tests, commonly referred to as Rapid Diagnostic Tests (RDTs). PfHRP3 is very similar to PfHRP2, with related alanine-histidine rich repeats and the same secretory signal. The histidine content ranges from approximately 28% in PfHRP3 to 34% in PfHRP2 [8].

The parasite expresses most of its genes as it invades and develops within the erythrocyte, with at least 60% of the genome transcriptionally active during the intraerythrocytic asexual cycle [15]. A number of housekeeping genes [16], [17], [18], [19], [20] including seryl tRNA synthase (*s-tRNA syn* or *tRNA*) and MAL13P1.209 60S ribosomal subunit protein L18 (*mal13*), are transcribed at a relatively constant rate across the asexual lifecycle of the 3D7 falciparum strain [15], [21], [22]. The relative abundance of many other mRNAs varies at different stages in the life cycle [15], [23], [24], [25], [26]. Overall mRNA levels vary significantly between different lifecycle stages and exhibit a moderately positive correlation with protein abundance [15], [23]. Groups of functionally related genes share common expression profiles [15].

A plausible hypothesis is that the amount of PfHRP produced by the parasite varies between different isolates, and as a consequence different amounts of this target protein would be available for detection by RDTs. PfHPR2 production and release has been characterised in a limited number of strains during the intraerythrocytic life cycle [8], [9], [11], [12], [27], [28]. However there is a paucity of data available on levels of intraerythrocytic transcription of *pfhrp2* and *pfhrp3* across the asexual cycle [29] and no detailed study has been undertaken on the variability of transcription and abundance of PfHRP across the asexual life cycle, and also between different parasite lines.

In this paper, we report the pattern of transcription of *pfhrp2* and *pfhrp3*, and of abundance of PfHRP protein, in different parasite strains, at several time points over the blood stage life cycle. Further, the potential impact that the variation in protein expression has on RDT detection sensitivity is investigated. Defining reasons for RDT failure is of significant public health importance and will contribute to improving diagnostics for falciparum malaria.

## Methods

### Sample material

Cryopreserved parasites originating from varied geographic areas (Table 1) were cultured *in vitro* [30] and synchronised repeatedly using 5% sorbitol solution [31] and once with the MACS^®^ Separation Column (Miltenyi Biotec USA) [32]. Seven lines were used to study the transcription dynamics of *pfhrp2* and *pfhrp3* through the erythrocytic life cycle (Table 1), where synchronised parasites (ring stage, approximately 5% parasitemia in 100 mL volume) were gently and thoroughly mixed and split into 10 equal aliquots of 10 mL each. Two aliquots were harvested immediately (time point 1), one flask for RNA isolation and another flask for protein isolation. The remaining aliquots were returned to culture and incubated at 37°C, then harvested in pairs at 12-hourly intervals (time points 2–5), to enable sampling at different stages of development over the intraerythrocytic life cycle. A further 10 parasite lines (Table 1) were used to compare the transcription levels of *pfhrp2* and *pfhrp3* at predominantly ring stage, the stage seen in patient blood. For RNA isolation, parasitized erythrocytes were pelleted, lysed using saponin (0.075%) in RNase-free tubes then frozen at −80°C. For protein isolation, culture supernatant was removed and red cell pellets were frozen at −80°C until protein extraction.

**Table 1:** Origin of parasite strains used in transcription of *pfhrp2* and *pfhrp3* and expression of PfHRP.

Isolate	Origin	Samples assayed	Remarks
Hb3	Honduras	Groups 1, 2, 4, 5	*pfhrp*3 negative control
Dd2	Thailand	Groups 1, 4, 5	*pfhrp2* negative control
D6	Africa	Groups 1*, 2, 4, 5	
K1	Thailand	Groups 1, 3, 4, 5	Identical *pfhrp2* sequence-GA3.
W2	Thailand	Groups 1, 3, 4	
PH1	Philippines	Groups 1, 4, 5	Identical *pfhrp2* sequence- PH3.
S55	Solomon Islands	Groups 1, 2, 3, 4	
N70	Solomon Islands	Group 1	Identical *pfhrp2* sequence-SJ44.
FCQ33	Papua New Guinea	Group 1	Identical *pfhrp2* sequence-FCQ41.
FCQ41	Papua New Guinea	Group 1	Identical *pfhrp2* sequence-FCQ33.
PH3	Philippines	Group 1	Identical *pfhrp2* sequence-PH1.
SJ44	Solomon Islands	Group 1	Identical *pfhrp2* sequence- N70.
GA3	Thailand	Group 1	Identical *pfhrp2* sequence-K1.
MCK	Malaysia	Group 1	
AN101	Papua New Guinea	Group 1	
FCR3	Africa	Group 1	
7G8	South America	Group 1	

*Assayed for protein level only.

### Confirmation of lifecycle stage

For each sample, the number and proportion of ring-stage parasites was determined by microscopically counting 500 infected erythrocytes. No gametocytes were observed in these cultures.

### RNA isolation

Total RNA was isolated from each parasite pellet using the NucleoSpin® RNA II Kit (Macherey-Nagel Germany), following the manufacturer's instructions. A second elution step was added to maximise yield as previously described [33]. All samples were treated with DNase, eluted in 60 µL RNase-free water; the eluted RNA was stored at −80°C.

### Real-time quantitative RT-PCR

Total RNA was reverse-transcribed into cDNA using SuperScript™ III Reverse Transcriptase (Invitrogen USA) and random hexamers. The real-time PCR primers were designed based on a *pfhrp2* fragment alignment of *P. falciparum* 3D7 (GenBank accession number BM275665), FCBR (GenBank accession X69922) and ITG2 (GenBank accession U69551). The forward primers for both *pfhrp2* and *pfhrp3* were designed to span both exon 1 and part of exon 2, with the reverse primer binding in exon 2. Primers for *pfhrp3* were designed using an alignment of ITG2 (GenBank accession U69552) and FCC1/HN (GenBank accession AF202093). Primer sequences are shown in Table 2.

**Table 2:** Primer sequences and real time PCR conditions used to quantify the transcription levels for *pfhrp2* and *pfhrp3* with reference housekeeping genes 3D7 *P. falciparum* gene family s-tRNA and 60S ribosomal subunit protein L18.

PCR conditions	Primer name	Sequence
95°C 15 mins, 1 cycle		Forward
40 cycles 95°C 30 sec,	*hrp2 rna 1*	TGTTAGATAACAATAATTCCGC
57°C 40 sec, 72°C 40 sec		Reverse
	*hrp2 exon 2 R1*	AGCATGATGGGCATCATCTA
95°C 15 mins, 1 cycle		Forward
40 cycles 95°C 30 sec,	*hrp3 rna 1*	TGTTAGATAACAATAACTCCGA
57°C 40 sec, 72°C 40 sec		Reverse
	*hrp3 exon 2 R1*	TGCATGATGGGCATCACCTG
95°C 15 mins, 1 cycle		Forward
40 cycles 95°C 30 sec,	*s-tRNA syn F*	AAGTAGCAGGTCATCGTGGTT
57°C 40 sec, 72°C 40 sec		Reverse
	*s-tRNA syn R*	TTCGGCACATTCTTCCATAA
95°C 15 mins, 1 cycle		Forward
40 cycles 95°C 30 sec,	*60S ribo L18 F*	ATTATCACATGGCCAATCACC
57°C 40 sec, 72°C 40 sec		Reverse
	*60S ribo L18 R*	CAATCTCTTATCATCTGTTATT

Real-time PCR conditions for *pfhrp2* and *pfhrp3* were optimised using *P. falciparum* 3D7 cDNA. The use of 150 nM primers per reaction and a 15 minute enzyme activation period on the MX4000 (Stratagene USA) gave the best reproducibility, and were used in all real-time PCR experiments for this study. Two housekeeping genes, PF07_0073 seryl tRNA synthase (*s*-*tRNA syn* or *trna*) gene and MAL13P1.209 (*mal13*), the 60S ribosomal subunit protein L18 were used as endogenous control transcripts to normalize mRNA levels [34].

All samples were run in triplicate using the ABsolute QPCR SYBR Green Mix (ABGene United Kingdom) according to the manufacturer's instructions. The following cycling conditions were used: 15 minutes at 95°C for initial denaturation and enzyme activation, followed by 40 cycles of 95°C for 30 seconds, 57°C for 40 seconds, and 72°C for 40 seconds, followed by a final extension of 57°C for 1 minute. The dissociation curve for *pfhrp2* gave a melting temperature at 76.7°C while for *pfhrp3* it was 77.8°C. Experiments where non-specific peaks in relation to the dissociation curves were observed were repeated. The mean Ct was determined and used in the ΔCt method [35] to calculate the amount of *pfhrp2* and *pfhrp3* relative to *s*-*tRNA syn* and *mal13*.

### Protein extractions

Pelleted infected red blood cells were thawed and resuspended in 1% Triton X-100 diluted with phosphate-buffered saline (PBS) and 1/1000 volume of a cocktail of proteinase inhibitors (Thermo Scientific USA). Samples were then frozen (−80°C) and thawed three times, with vortexing every 15 minutes. After protein solubilization the solution was spun at 15 000 rpm for 30 minutes at 4°C and the supernatant removed for use in protein experiments.

### Western blot analysis

A 40 µL supernatant aliquot was diluted 1∶5 with PBS-T (PBS- Tween 20, 0.05%) to reduce interference from residual hemoglobin. One third volume of 3×SDS sample loading buffer was added to the extracts, and boiled for 10 minutes prior to loading on an SDS-page gel (NuPage 10% Bis-Tris Gel, Invitrogen USA), run for 1 hour at 60 V then 1.5 hour at 150 V. Proteins were then transferred from the SDS-page gel to a 0.45 µm PVDF membrane (Invitrogen USA) at 100 V for 1.5 hours. Following transfer, the membrane was blocked overnight in 5% skim milk powder dissolved in 100mL PBS-T. The membrane was then incubated with the primary antibody, PTL3 (kindly provided by Dr Martin Bubb, National Bioproducts Institute, South Africa) at 1∶5000 dilution. Following washing in PBS-T, the membrane was incubated with an anti-mouse polyvalent immunoglobulin-alkaline phosphatase conjugate (Sigma USA) at 1∶5000 dilution. The signal was detected by CDP Star chemiluminescent substrate (Roche Germany) and exposed to imaging films.

### Enzyme-Linked Immunosorbent Assay

A PfHRP2 quantitative antigen-capture ELISA (Bioline SD Malaria Antigen Pf, Standard Diagnostics Korea) was used according to the manufacturer's instructions. Positive and negative control wells were included to quantitate protein levels for all samples. 3D7 culture supernatant containing a known concentration of PfHRP2 was used for standard curve construction. All samples were tested as undiluted, 1/100, 1/200 and 1/300 dilutions and run in duplicate. Absorbance was measured at 450 nm and 650 nm using a microplate spectrophotometer (SpectraMax Molecular Devices USA).

The software package SoftMax Pro software (Molecular Devices USA) was employed to quantify the protein levels in ng/mL. Samples whose concentration fell outside the standard curve were further diluted and the test repeated. Calculated PfHRP2 levels were then normalised against the samples own *trna* transcription value to account for potential differences in the number of parasites in the sample. The *trna* values from the transcript dataset for the K1 parasite were then used for this normalisation: normalised PfHRP2_sample parasite_ = PfHRP2_sample parasite_×(*trna*_sample parasite_/*trna*_K1_).

### Rapid Diagnostic Tests

To investigate whether differences in PfHRP protein level results in a difference in the detection limit on RDTs, the ring-stage protein sample (80–100% ring) from parasite strains K1, D6 and Hb3 were tested without dilution, then at 1/100, 1/200, and 1/300 dilutions in 1×PBS, on three RDTs (ICT Malaria, ICT Diagnostics South Africa; First Response Malaria Antigen Combination Test, Premier Medical Corporation India; SD Malaria Rapid Test, Standard Diagnostics Korea).

The experiment was then repeated using the same number of intact parasitized red blood cells of the three parasite lines at seven doubling dilutions (15 000 P/µL serially diluted down to 10 P/µL) at 50% hematocrit. The RDTs were read using the WHO Colour Intensity chart for RDTs with a scale of 0-4, based on the intensity of the band colour, 4 being the strongest band colour, 3 moderate colour, 2 weak colour, 1 faint colour, and 0 negative (Dr D. Bell, WPRO-WHO, 2004, unpublished).

### Statistical analysis

Spearman Rank Correlation using GraphPad PRISM software (GraphPad Software, La Jolla USA) was applied to assess the relationship between transcript and parasite stage, protein and parasite stage, and the relationship between transcription and expression.

## Results

### Grouping parasites by developmental stages

Preliminary experiments indicated that the different strains of *P. falciparum* to be characterised in this work have different asexual replication times. To compare levels of transcription of *pfhrp2* and *pfhrp3* at the well-recognized asexual stages, we grouped parasites based on the proportion that were ring-stage at different times across the lifecycle. For 5 of the 7 lines studied (HB3, Dd2, W2, K1 and PH1, Table 1) the parasites present at time points 1, 2 and 5 were predominantly rings, trophozoites at time point 3, and schizonts at time point 4. Parasites returned to ring stages at time point 5 corresponding with the start of a new cycle. In contrast, the lifecycle of the D6 and S55 parasite lines was shorter, with parasites being predominantly rings by time point 4. This indicates that different parasite strains have different asexual replication times. To reflect transcriptional changes for *pfhrp2* and *pfhrp3* at different asexual stages, we grouped parasites based on the proportion of parasites that were ring-stage: Group 1, 80–100%, Group 2, 60–79%, Group 3, 40–59%, Group 4, 20–39% and Group 5, 0–19%. After grouping, some parasite lines have samples representing 4 groups and some have samples representing only 3 groups (Table 1). This is because of differences in the age of rings at the time when experiment started and differences in the growth rate of different lines.

### Transcription of *pfhrp2* and *pfhrp3* peaks at ring stage

**Figure 1:**
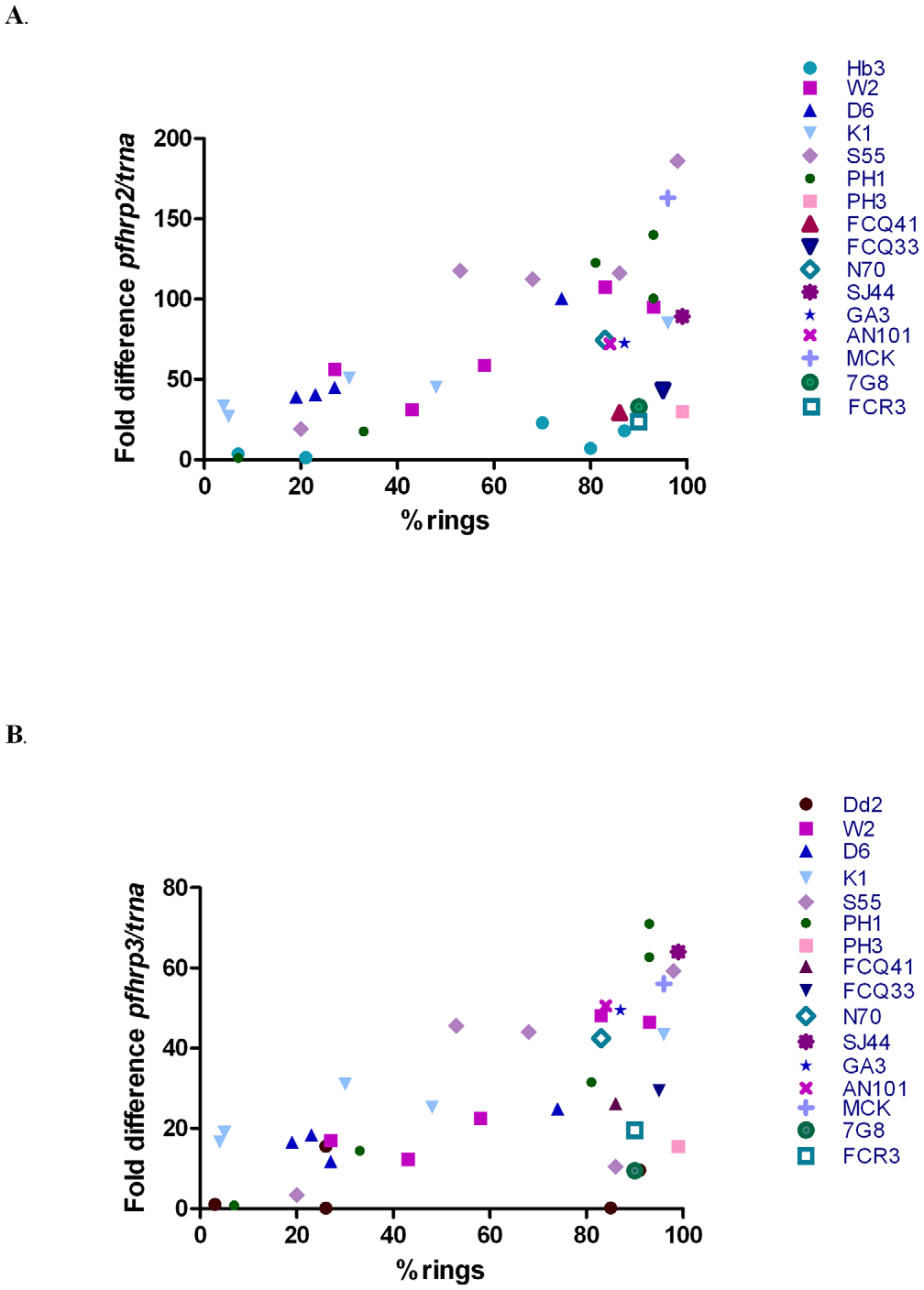
Transcription of *pfhrp2* (A) and *pfhrp3*(B) normalised to *trna* over the intraerythrocytic life cycle of parasites (with varying proportions of ring stage).

Using quantitative real-time PCR, transcription of both *pfhrp2* and *pfhrp3* was observed to peak at ring stage (Figure 1A and 1B). It should be noted that transcription level of *pfhrp2* was not determined for Dd2 because it lacks the *pfhrp2* gene, and that transcription levels of *pfhrp2* and *pfhrp3* were not determined for the group 1 sample of D6 because the RNA was degraded (D6 had a Group 1 protein sample but no Group 1 mRNA sample). A significant positive correlation was observed between the proportion of rings and the level of transcription for both *pfhrp2* and *pfhrp3* (P < 0.01) (Figure 1A and 1B). This was a consistent finding irrespective of whether the transcription levels of *pfhrp2* and *pfhrp3* were normalised against *trna* (Figure 1A and 1B) or against *mal13* (Figure S1A and S1B).

### Transcription of *pfhrp2* and *pfhrp3* varies between parasite strains at different lifecycle stages

**Figure 2:**
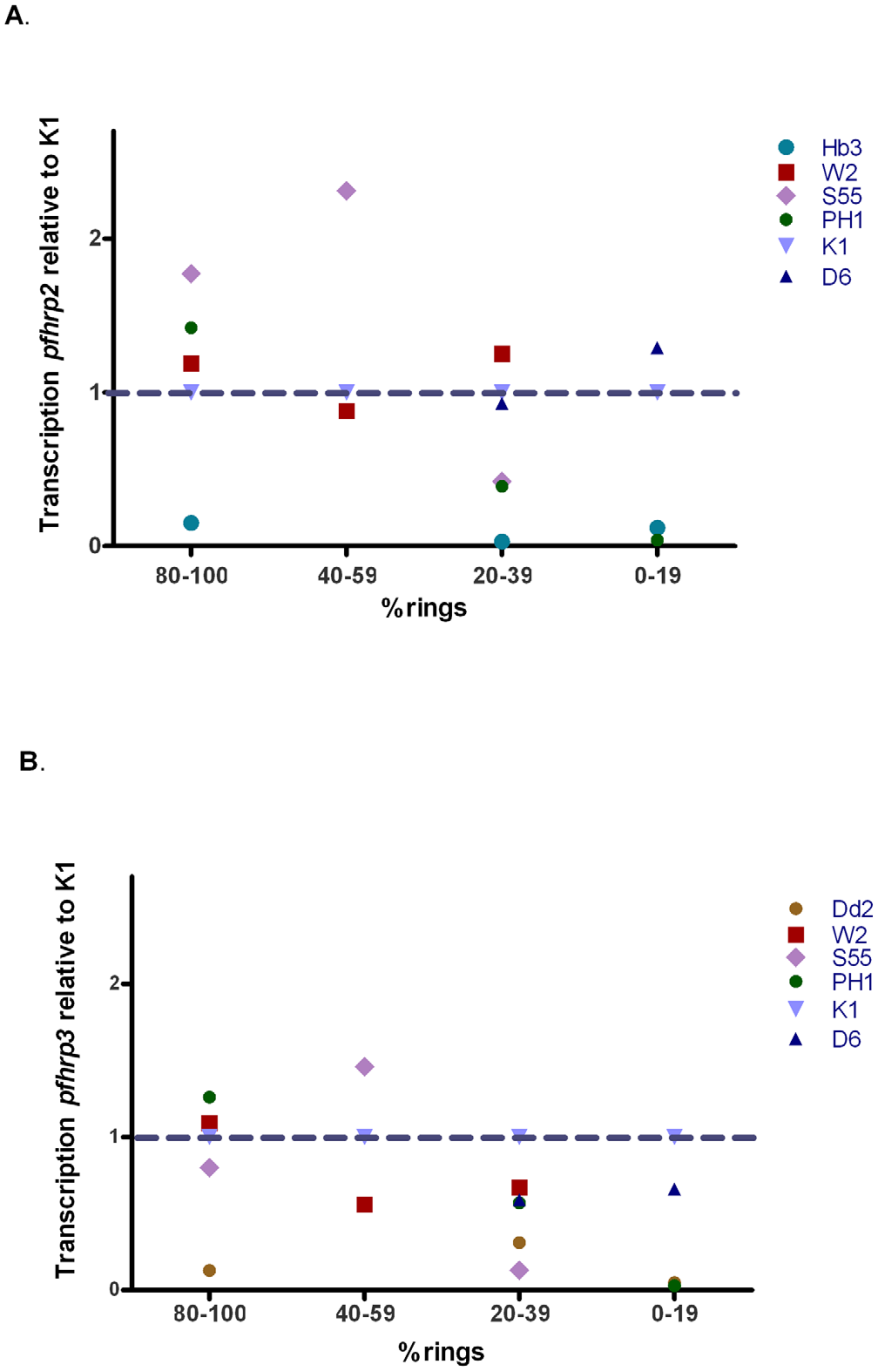
Quantity of *pfhrp2* (A) and *pfhrp3* (B) transcripts in 5 *P. falciparum* lines over the intraerythrocytic life cycle (with varying proportion of ring stage) relative to K1.

The level of transcription of *pfhrp2* and *pfhrp3* relative to the K1 control parasite strain varied over the course of the intraerythrocytic life cycle in all strains studied (Figure 2A and 2B). Levels were also observed to vary between strains. However, the rank order of the levels of transcription between strains varied at different time points. For example, S55 strain ranked the highest in quantity of *pfhrp2* transcript when the proportion of rings was between 40 to 100%, but ranked lower when the proportion of rings fell below 40%.

### Transcription of *pfhrp2* is higher than *pfhrp3*

**Figure 3:**
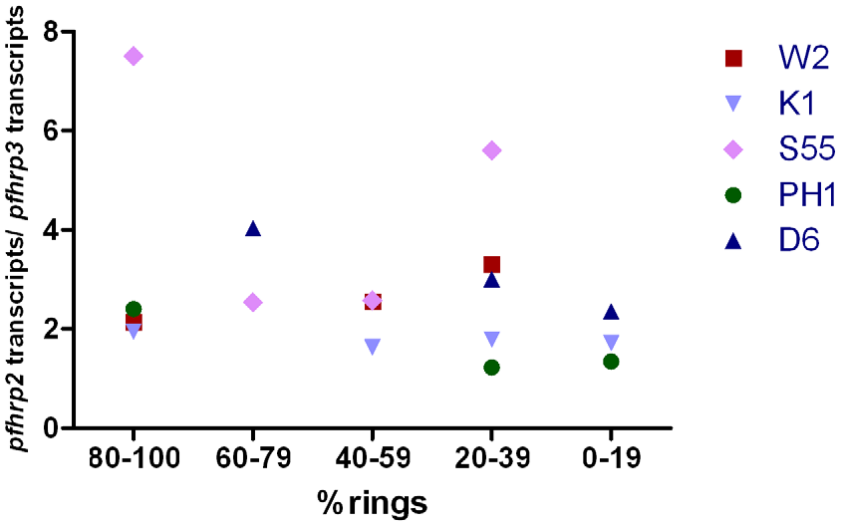
Quantity of *pfhrp2* transcription relative to *pfhrp3* transcription in 5 parasite strains at varying proportion of ring stage.

Levels of transcription of *pfhrp2* were consistently higher than for *pfhrp3* in all parasite lines and at all time points tested. This finding was true irrespective of the housekeeping gene used to normalise the data. Figure 3 shows the data normalised to *trna* gene.

### Levels of transcription of *pfhrp2* vary between strains at ring stage

**Figure 4:**
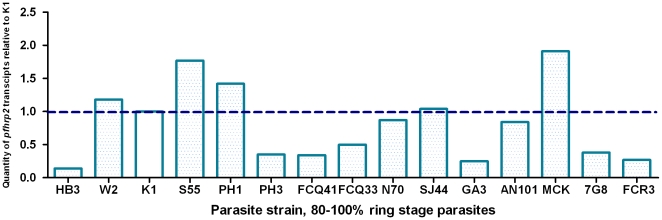
Quantity of *pfhrp2* transcripts (relative to K1) in samples with 80–100% ring stage for 15 *P. falciparum* lines.

Levels of *pfhrp2* transcripts at 80–100% ring stage were compared for 15 strains against K1 (Figure 4). The ratio of transcription levels relative to K1 control parasite varied from a minimum ratio of 0.15 (Hb3) to a maximum of 1.9 (MCK). The experiment including synchronisation, culture and quantification of transcripts was repeated for 5 parasite lines at ring stages. The differences in transcription measurements of the two experiments were < 5.3%. As the sequence of the *pfhrp2* gene varies significantly between strains [7], a separate analysis of transcription levels for parasite strains with the same *pfhrp2* sequence was undertaken. Of interest, differences in transcription were marked between some paired strains: PH1 and PH3 (1.4 vs 0.4), K1 and GA3 (1.0 vs 0.3), and moderate between SJ44 and N70 (1.1 vs 0.9), FCQ33 and FCQ41 (0.5 vs 0.3).

### Transcription of pfhrp2 and pfhrp3 is reduced when one of the genes is absent

*Pfhrp3* is absent in the *P. falciparum* laboratory clone Hb3 [36]. Of interest, we observed that the level of transcription of *pfhrp2* in this parasite line was lower compared to all other strains tested. In other words, no compensatory increase in *pfhrp2* transcription was observed when *pfhrp3* was absent. Similarly, the parasite line Dd2 lacks *pfhrp2* [37]. We likewise observed that transcription of *pfhrp3* in this line was the lowest of those tested with no compensatory increase to account for the lack of *pfhrp2.*

### Levels of histidine-rich proteins

Monoclonal antibodies raised against PfHRP2 have been shown to cross-react with PfHRP3 [8]. Thus, although our experiments were designed to measure the level of PfHRP2 protein, it should be noted that PfHRP3 was also likely to contribute to the result. As no PfHRP3 specific antibody ELISA was available, we were unable to investigate the relative contribution of PfHRP2 and PfHRP3 in the PfHRP quantitative ELISA.

**Figure 5:**
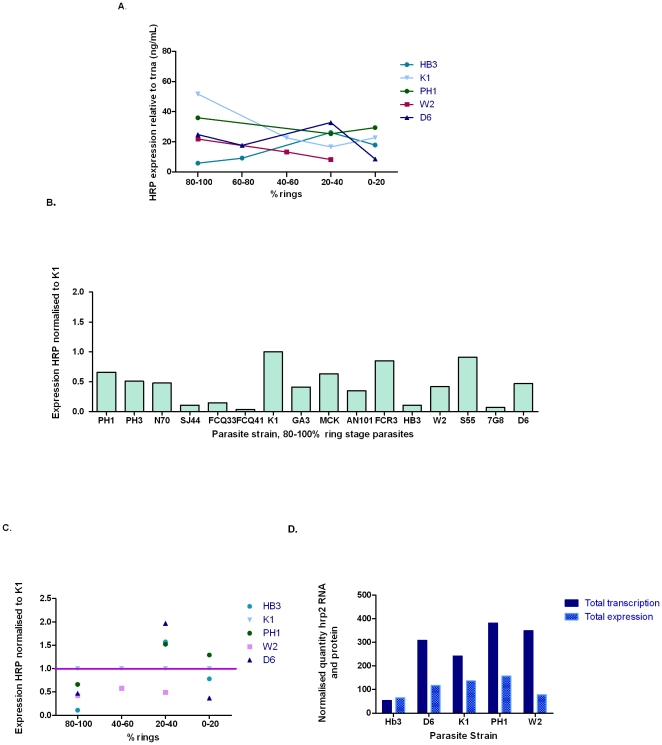
Expression of PfHRPs in different *P. falciparum* lines over the intraerythrocytic life cycle. (A) Quantity of HRP protein expression in parasites with varying proportion of ring stage of 5 *P. falciparum* lines. (B) Protein expression values relative to K1 for PfHRP in samples with 80–100% ring stage for 15 *P. falciparum* lines. (C) Quantity of HRP protein expression in parasites with varying proportion of ring stage for 5 *P. falciparum* lines normalised to K1. (D) Comparison of total *pfhrp2* transcription and total expression of PfHRP across the intraerythrocytic life cycle for 5 parasite lines.

Western blot analysis confirmed the presence of PfHRP2 at all time points examined across the asexual life cycle (Figure S2). When levels of PfHRP protein were measured by ELISA over the intraerythrocytic life cycle, results demonstrate that the protein levels were relatively constant throughout the erythrocytic cycle compared to transcription levels in 5 lines tested (Figure 5A). When levels in 16 strains were measured at 80–100% ring stage, there was a considerable difference in protein expression between strains with a maximum of 24 fold (1.20 in S55 vs. 0.05 in FCQ41) difference in PfHRP levels (Figure 5B). When levels of PfHRP in parasite strains with identical *pfhrp2* sequence were compared at predominant ring stage (Group 1), a 1–4 fold difference in protein levels was observed between the paired strains (Figure 5B). Three parasite lines were cultured, synchronised and harvested again to repeat the experiment. Measured levels of protein expression between the two experiments differed by a maximum of 22% for the 3 parasite lines tested.

In contrast to transcription, protein levels did not appear to be related to the number of ring stage parasites (P > 0.05, Figure S3). The intraerythrocytic lifecycle stage at which the highest level of protein was present varied across strains (Figure 5A). Expression of PfHRP was observed to vary in different stages relative to the K1 control parasite (Figure 5C).

### Correlation between transcription level of *pfhrp2* and abundance of PfHRP

No significant correlation was observed between abundance of *pfhrp2* transcripts and the level of PfHRP protein (P > 0.05, Figure 5D). For a sample size of five isolates where both transcription and protein abundance across the intraerythrocytic life cycle were determined, our data were not conclusive. A larger sample size may provide a better statistical analysis for examining the relationship between transcription and expression for this gene and protein.

### Higher protein levels result in a lower detection limit on RDTs

The K1 and D6 parasite lines had a higher level of PfHRP protein at ring-stage than the Hb3 parasite line. These three parasite lines were used to test the sensitivity of three brands of malaria RDTs. When the parasite protein extracts were tested with the RDTs, K1 and D6 showed good detection sensitivity down to the maximum dilution of 1/300, while Hb3 tested negative when diluted below 1/100. The results confirm that samples with higher protein levels test positive in RDTs at higher dilutions than those with lower levels of protein.

The experiment was repeated using an identical number of parasitized blood cells of the cultured lines. D6 returned strong positive results at all seven dilutions tested at 15 000 P/µL serially diluted down to 10 P/µL, with an intensity score of 4 based on the WHO RDT Colour Intensity Chart (Dr D. Bell, Western Pacific Regional Office of the World Health Organization, WPRO-WHO, 2004, unpublished). K1 returned strong positive results of 4 for all dilutions except the final dilution where the band intensity was slightly weaker at a score of 3. Hb3 worked only weakly until the second dilution, 10 000 P/µL with a score of 2.

## Discussion

The purpose of this study was to investigate whether the transcription of the *pfhrp2* and *pfhrp3* genes, and the levels of the corresponding protein PfHRP, vary between different parasite strains, and whether these variations influence the detection sensitivity of malaria RDTs. Our data indicate that *pfhrp2* and *pfhrp3* transcription is highest at the ring stage of the intraerythrocytic life cycle, a finding that agrees with the transcription profiles for these genes using microarray analysis for the 3D7 strain which is available in the PlasmoDB database [29]. In this study we have shown that transcription levels of *pfhrp2* vary widely between geographically variant strains of the parasite, even for strains with the same *pfhrp2* sequence and also between strains with different *pfhrp2* sequence.

Control of transcription is believed to be under the influence of various regulatory systems, many of which are incompletely understood in *Plasmodia* [24], [38].

It is clear that transcription patterns vary between genetically distinct parasites [39]. The 5′ untranslated region of genes (UTR) may contain regulatory elements that interact with promoter region elements to regulate transcription, or influence mRNA stability or survival. The 3′ UTR may also have an important role in regulation of gene function [40]. Although not investigated in this study, different parasite lines with different *pfhrp2*, or even identical coding sequences may differ in their 5′ UTR and 3′ UTR sequences, resulting in different levels of transcription.

Epigenetic mechanisms may also significantly affect transcription [41]. Changes in chromatin structure may profoundly influence the relationship between promoter and transcription factors [38]. Nucleosome-free regions found at transcription start sites and core promoters are strongly associated with high levels of gene expression in intraerythrocytic stages [42]. It is therefore possible that epigenetic factors play a role in *pfhrp2* and *pfhrp3* transcription.

It should be noted that the levels of *pfhrp2* and *pfhrp3* transcripts and PfHRP protein measured in this study constitute the amounts of transcript or protein present at that timepoint, reflecting the combined result of transcription and degradation of the target. For transcripts, post-transcriptional control mechanisms including mRNA stability and decay may contribute significantly to the variation in the amount of transcripts present [43]. mRNA translation efficiency can also be influenced by a range of factors. Histone 396–494 of *pfhrp3* may play a role in mRNA stability [44]. Variation in binding factor sequences in the 5′ and 3′ UTR may also exert a significant influence on mRNA stability and translation. Likewise, changes to the transcription start site may regulate the translation efficiency of a given gene [40].

In all strains tested in this study, we observed that the level of *pfhrp2* transcripts was always higher than that of *pfhrp3*. This could be a consequence of the promoter for *pfhrp2* being stronger, or as a result of a slower decay of *pfhrp2* transcripts. A 5′ flanking region of *pfhrp3* has been extensively used in transfection studies as a promoter for reporter gene expression, paired with a 3′ flanking region of *pfhrp2* as a terminator sequence [45], [46]. However, there is no report of using the *pfhrp2* promoter for transfection, thus precluding a comparison of the two promoters. In an earlier study, levels of PfHRP2 to PfHRP3 were compared using immunochemical methods, with results indicating that PfHRP3 protein levels were lower than PfHRP2 [8].

The *pfhrp2* and *pfhrp3* genes are structurally very similar, with regions flanking the tandem repeats, including untranslated regions, showing up to 90% homology [8], [10]. Both genes share many motifs and the same secretory leader. Exon 2 of both *pfhrp2* and *pfhrp3* genes encode the histidine-rich amino acid repeats beginning 75–90 nucleotides downstream from the start [13]. Given the similarity of the two genes, it is plausible that their gene products perform similar functions, and that their transcription may be controlled by a common regulatory mechanism. However, we observed that *pfhrp2* transcription was lower when *pfhrp3* was absent and vice versa. If the products of these two genes have a similar function, it would be expected that one would compensate for the absence of the other. From our data, it may be suggested that these genes have a non-overlapping function. *pfhrp2* and *pfhrp3* are not linked, occurring as single copy genes on separate chromosomes [36]. Parasites lacking either one gene or both genes have been reported in both laboratory lines [47] and in clinical isolates in South America [48], suggesting these genes are not essential for the survival and transmission of the parasite in the human host.

The variation in transcription we observed was also reflected by differences in the protein expression levels. Although PfHRP protein expression was more constant throughout the intraerythrocytic life cycle in 5 lines tested, wide variations in the level of protein were observed between 16 strains at ring stage, the stage circulating in patient blood, with up to a 24 fold difference observed between strains. The results are in agreement with RDT results observed using patient samples at 200 P/µL recently reported from Colombia [49].

Importantly, we have shown that parasite strains with higher protein levels have a lower detection limit on commercial RDTs compared to those with lower protein levels. The same phenomenon was observed when the number of parasites was controlled. Although the quantity of HRPs in D6 was less than that in K1, RDTs used to test these lines were strongly positive. When compared to the Hb3 result, which had a low protein level and was not detected at low concentrations, a threshold is observed, below which the level of protein affects RDT sensitivity. Parasites with low levels of protein will be difficult to detect at low parasitemia (≤200 P/µL). This provides further evidence that the level of PfHRP protein is a major determinant of the detection sensitivity of RDTs, and therefore that such variations in protein abundance could contribute to variation in the performance of PfHRP2- detecting RDTs. This consideration is particularly important for testing of patients at low parasitemia, where parasites with lower protein levels may return negative results on malaria RDTs. Likewise, low parasite protein expression may also explain why some RDTs return a negative result at relatively high (>1 000 P/µL) parasitemia levels. An increased understanding of the factors influencing RDT performance will likely help to improve the performance and use of such malaria RDTs that are of significant public health importance.

It should be pointed out that the parasite lines used in this study have all been adapted to *in vitro* culture conditions. Although neither *pfhrp2* nor *pfhrp3* genes were deleted during culture adaptation, it is unknown whether levels of transcription of these genes, and expression levels of the encoded proteins were affected during the adaptation. Further work using these methods to quantify transcription and expression on additional parasite isolates, and especially testing of field isolates, to determine the extent of variation, and the proportion of parasite isolates that produce low level HRPs would strengthen this observation. The impact of variation in protein level on RDT detection sensitivity in patients also requires further study. Important experimental constraints impede the measurement of PfHRP in field samples. These include the lack of synchrony and inability to control parasitemia levels.

### Conclusions

We have shown here that transcription of *pfhrp2* and *pfhrp3* and expression of PfHRP protein levels varies between laboratory strains of the parasite, and demonstrated that the variation in protein levels results in differences in RDT detection thresholds. The outcome of this study provides a possible explanation for reported variation in sensitivity of PfHRP2-detecting RDTs and will assist research aimed at improving malaria RDTs.

## Supporting Information

Figure S1 **Transcription of** ***pfhrp2*** **(A) and** ***pfhrp3*** **(B) normalised to** ***mal 13*** **over the intraerythrocytic life cycle (with varying proportion of ring stage).**
(TIF)

Figure S2 **Western Blot of PfHRP2 for 5 time points, D6 line.**
(TIF)

Figure S3 **Plot of PfHRP expression level against the proportion of ring stage parasites.**
(TIF)
