# Supplementary figures and images for: Transcription and Expression of Plasmodium falciparum Histidine-Rich Proteins in Different Stages and Strains: Implications for Rapid Diagnostic Tests
Source: PLoS One. 2011 Jul 22;6(7):e22593. doi: 10.1371/journal.pone.0022593 (PMC3142190; doi:10.1371/journal.pone.0022593)

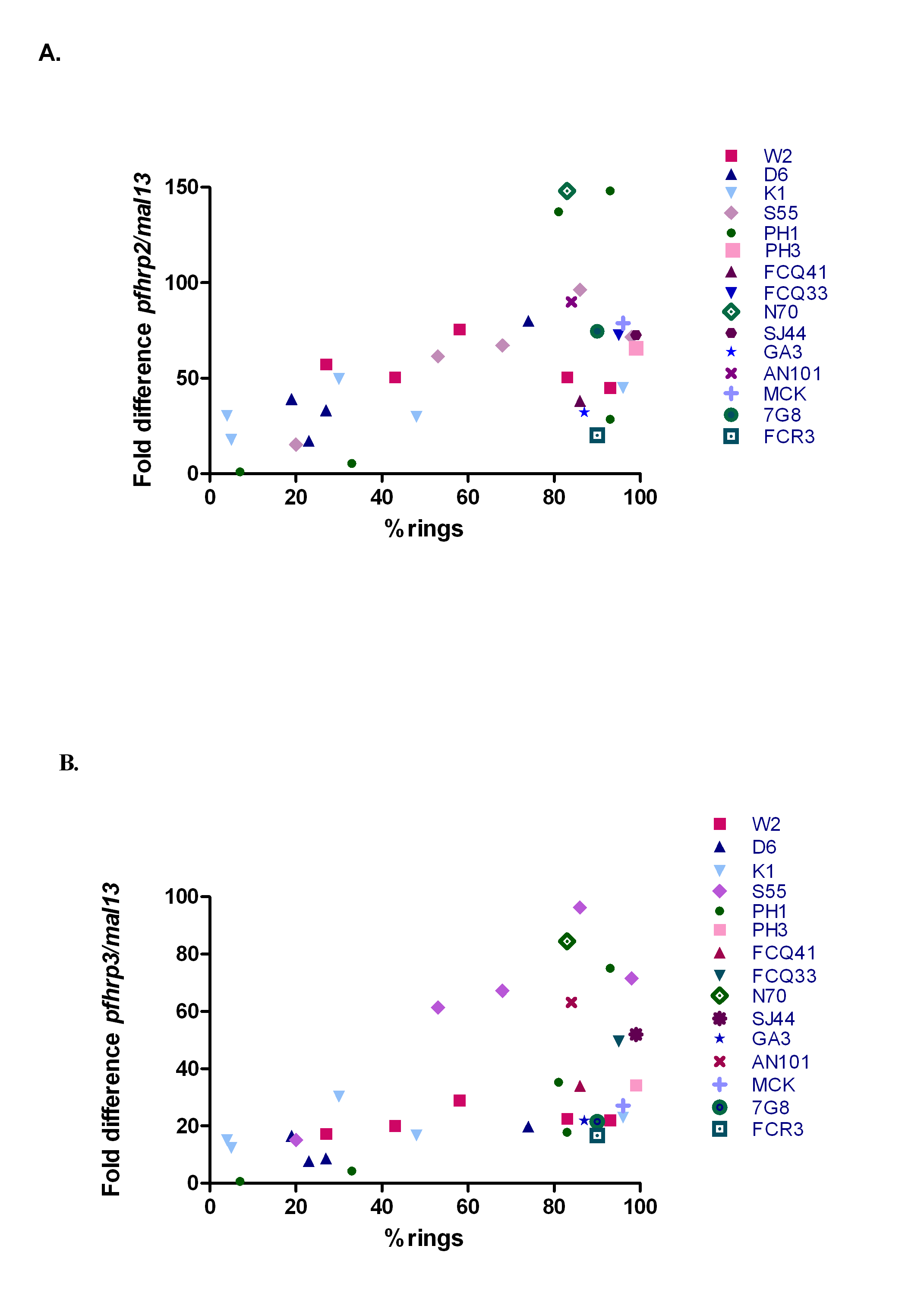

Supplement: Figure S1 — Transcription of pfhrp2 (A) and pfhrp3 (B) normalised to mal 13 over the intraerythrocytic life cycle (with varying proportion of ring stage). (TIF) [file pone.0022593.s001.tif]

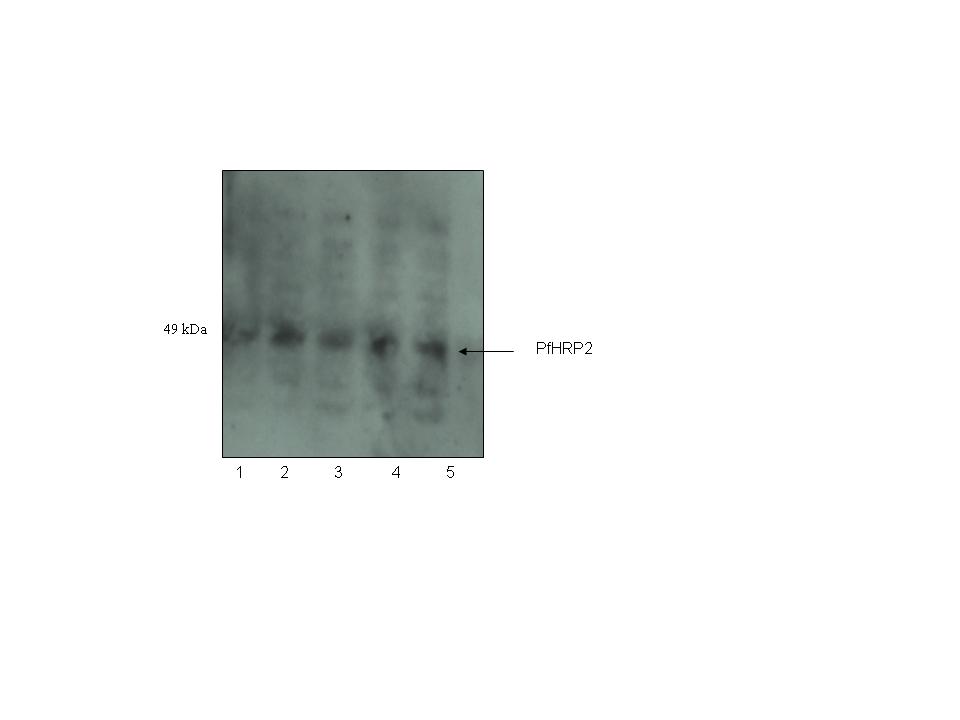

Supplement: Figure S2 — Western Blot of PfHRP2 for 5 time points, D6 line. (TIF) [file pone.0022593.s002.tif]

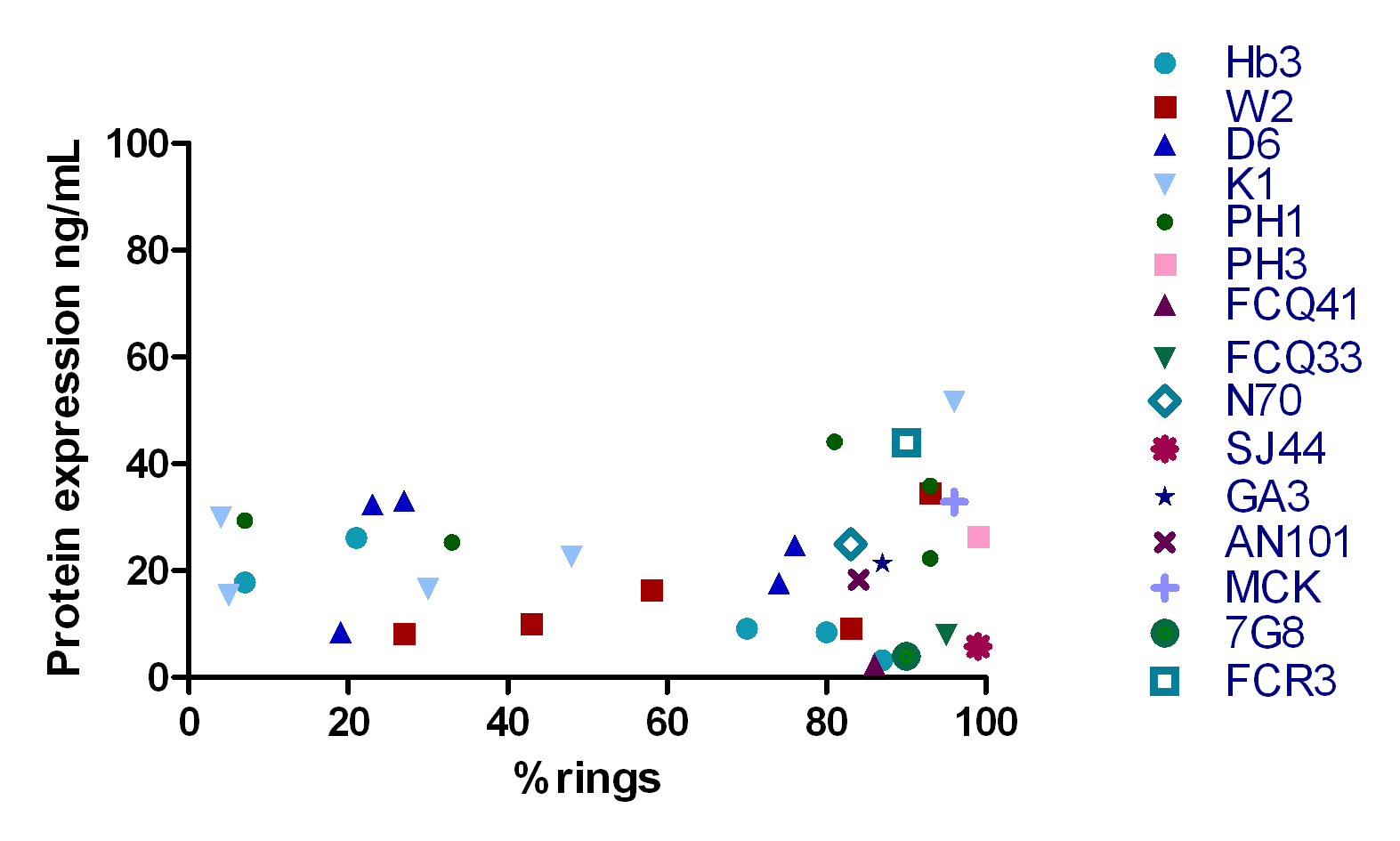

Supplement: Figure S3 — Plot of PfHRP expression level against the proportion of ring stage parasites. (TIF) [file pone.0022593.s003.tif]
